# Essential Oils: Recent Advances on Their Dual Role as Food Preservatives and Nutraceuticals against the Metabolic Syndrome

**DOI:** 10.3390/foods12051079

**Published:** 2023-03-03

**Authors:** Emily L. Chávez-Delgado, Daniel A. Jacobo-Velázquez

**Affiliations:** 1 Tecnologico de Monterrey, Escuela de Ingenieria y Ciencias, Ave. General Ramon Corona 2514, Zapopan 45138, Jalisco, Mexico; 2 Tecnologico de Monterrey, The Institute for Obesity Research, Ave. General Ramon Corona 2514, Zapopan 45201, Jalisco, Mexico

**Keywords:** essential oils, metabolic syndrome, diabetes, obesity, neuroprotection, antioxidant, antimicrobial, nanoencapsulation

## Abstract

Essential oils (EO) are compounds synthesized by plants as secondary products and are a complex mixture of volatile molecules. Studies have demonstrated their pharmacological activity in the prevention and treatment of metabolic syndrome (MetS). Moreover, they have been used as antimicrobial and antioxidant food additives. The first part of this review discusses the role of EO as nutraceuticals to prevent metabolic syndrome-related disorders (i.e., obesity, diabetes, and neurodegenerative diseases), showing results from in vitro and in vivo studies. Likewise, the second part describes the bioavailability and mechanisms of action of EO in preventing chronic diseases. The third part presents the application of EO as food additives, pointing out their antimicrobial and antioxidant activity in food formulations. Finally, the last part explains the stability and methods for encapsulating EO. In conclusion, EO dual role as nutraceuticals and food additives makes them excellent candidates to formulate dietary supplements and functional foods. However, further investigation is needed to understand EO interaction mechanisms with human metabolic pathways and to develop novel technological approaches to enhance EO stability in food systems to scale up these processes and, in this way, to overcome current health problems.

## 1. Introduction

Obesity is the leading risk factor for metabolic syndrome (MetS) due to energetic imbalance; this can cause impaired glucose tolerance, insulin resistance, type 2 diabetes, dyslipidemia, hypertension, and a chronic proinflammatory state [1,2]. Non-pharmaceutical alternatives, such as nutraceuticals, phytotherapy, and functional foods, have been explored to prevent and treat common diseases, and their use is increasing in the public domain by up to 50–70% [2]. Nevertheless, their long-term safety, efficacy, and dose schemes are being actively researched [3,4].

Essential oils (EO) are composed of a mixture of natural, volatile, aromatic compounds characterized by a strong odor and are produced as secondary metabolites by aromatic plants in different plant organs, including buds, flowers, seeds, leaves, roots, fruits, wood, twigs or bark [5,6,7].

Extraction methods are divided into conventional and non-conventional techniques. Common conventional techniques include hydrodistillation, Soxhlet extraction, water distillation, steam distillation, and organic-solvent extraction. Non-conventional extraction processes include ultrasound-assisted extraction (UAE), microwave-assisted extraction (MAE), high-pressure (HP), pressurized liquid extraction (PLE), negative pressure cavitations-assisted extraction (NPCE), subcritical water extraction (SWE), supercritical fluid extraction (SFE), enzyme-assisted extraction (EAE), pulsed electric field-assisted extraction (PEF), and accelerated solvent extraction (ASE). These extraction techniques and non-thermal and are categorized as “green”, since they use lower amounts of solvents, energy, and time, giving higher yields compared to conventional methods [6,8].

Organic compounds present in EO vary between 20–200 different types, with the vast majority present in traces, although two or three of these compounds are the most representative ones (20–70%) and are thought to be responsible for the biological activities of the EO [6,7]. Despite that, terpene hydrocarbons are the primary chemical group found in EO [6]. Terpene hydrocarbons are classified as monoterpenes (C_10_), which are the major constituents of EO; sesquiterpenes (C_15_); diterpenes (C_20_); terpenoids (oxygenated terpenes); and aromatic compounds, such as phenylpropanoids, derived from phenylpropane [7].

Traditional medicinal herbs and their derived EO are phytochemical-rich sources of health-promoting bioactive compounds [9]. As nutraceuticals, some reported health benefits of EO include antioxidative, antimicrobial, antitumor, anticarcinogenic, anti-inflammatory, antiatherosclerosis, antimutagenic, antiplatelet aggregation, and angiogenesis inhibitory activities [1,3,4,6,7,9].

Nowadays, EO have been used in the food industry as food additives due to their antioxidant and antimicrobial properties. A large variety of EO from different plants have been incorporated into food systems, such as basil, chamomile flowers, cardamom seeds, and rosemary [10].

There is active research in the study of EO as natural potential candidates to prevent and treat MetS. Their incorporation in proper food vehicles to achieve this goal is a new and interesting approach that deserves particular interest. For this reason, several studies focused on these fields are recapitulated herein. However, to the best of our knowledge, few or no studies are focused on using EO as nutraceuticals and food additives to treat metabolic and non-communicable diseases. Therefore, this review is intended to give a new approach for the production of functional foods using EO as promising molecules.

This review is divided into four main sections; the first part discusses the effects of EO in MetS and its comorbidities, specifically in obesity, diabetes, and neurodegenerative diseases, including in vitro and in vivo studies and clinical trials. The second section describes the bioavailability and EO mechanisms of action of their common administration routes (i.e., oral, dermal, and pulmonary administration). The third part presents the incorporation of EO as food additives and discusses current applications in food systems as antimicrobial and antioxidant agents. Finally, the last part focuses on the stabilization and common methods for encapsulating EO in other to preserve their bioactivity and how they could be incorporated into food matrixes.

## 2. Materials and Methods

In this review article, EO bioactivities and additive properties reported were searched in several databases such as Elsevier, Google Scholar, PubMed, and SpringerLink via Tecnológico de Monterrey library system. In these databases, keywords were: “essential oils, anti-obesity, anti-diabetes, metabolic syndrome, adipogenesis inhibition, postprandial hyperglycemia control, neuroprotection, microencapsulation, and antimicrobial and antioxidative properties”.

Research and review articles were selected based on the use of EO in controlling these pathologies and their incorporation into food matrixes. In the case of anti-obesity, anti-diabetes, and neuroprotection activities, those studies focused only on individual or majority components of EO were excluded since it was intended to show the bioactivity of EO per se.

Because this field has been relatively little explored, we aimed to explore extensively what has been reported over the past 18 years (2005–2023).

## 3. Use of EO for Metabolic Syndrome-Related Disorders Management

MetS is a combination of metabolic disorders that comprises central obesity, insulin resistance, hypertension, and atherogenic dyslipidemia. These factors propitiate chronic inflammation, leading to cardiovascular disease (CVD) development. Because obesity rates have been increasing worldwide, MetS has become highly relevant; thus, early prevention and treatment are crucial factors in decreasing mortality rates [1,11]. It has been reported the use of EO for treating obesity and diabetes. The following sections present in vitro and in vivo studies and clinical trials regarding the use of EO for preventing and treating metabolic syndrome-related disorders.

### 3.1. Anti-Obesogenic Potential

Adipocytes are cells in charge of maintaining energetic homeostasis. These cells are present in white adipose tissue (WAT) and brown adipose tissue (BAT). WAT is important in energy storage, while BAT uses energy to produce heat. Morphologically, WAT is characterized by one single lipid droplet, whereas BAT comprises many multilocular liquid droplets and mitochondria [12].

Excessive fat accumulation results in inflammation and oxidative stress in adipose tissue as a result of a constant elevation of plasma-free fatty acids (FFAs) caused by a growing release from enlarged adipose tissue that activates and upregulates the expression of several proinflammatory cytokines such as tumor necrosis factor-α (TNFα), interleukin (IL)-1β and IL-6, which aggravates metabolic alterations [13,14]. For instance, the constant liberation of FFAs promotes the synthesis of very low-density lipoprotein, cholesterol, and gluconeogenesis in the liver, thus provoking impaired insulin signaling and glucose metabolism and, by this process, causing insulin resistance [15].

Fat homeostasis is driven by two principal metabolic pathways: lipogenesis and lipolysis. The former is accountable for packaging esterified triglycerides in the liquid droplet when there is an excess of nutrients, thus expanding adipose tissue, which may play a central role in obesity comorbidities. At the same time, the latter hydrolyze lipid triglycerides into glycerol and three fatty acids [16,17,18].

Lipogenesis is driven by two principal transcriptional regulators, the Sterol Response Element Binding Protein 1c (SREBP1c) and the Carbohydrate Response Element Binding Protein (ChREBP). The activation of both pathways is due to increased insulin signaling in response to high glucose levels [14]. Lipogenesis inhibition is a promising strategy to prevent fat storage in adipocytes; therefore, WAT and BAT are crucial targets for obesity treatment and related diseases [12].

Adipocyte differentiation, also known as adipogenesis, is a process in which preadipocytes are transformed into mature adipocytes [14]. It plays a vital role in regulating obesity; for instance, combined with adipocyte hypertrophy, it is is the primary mechanism leading to this disorder [19,20]. In vitro and in vivo experiments on the anti-obesity potential of different EO are presented in Table 1.

Regarding adipogenesis, this process compromises several steps guided by different transcription factors regulating adipogenic gene expression. There are two important families, the CCAAT/enhancer-binding proteins (C/EBPs) and the peroxisome proliferator-activated receptors (PPARs). Adipogenesis initiates with the expression of C/EBPβ and C/EBPδ, which in turn activates C/EBPα and PPARγ mRNA. These molecules trigger the transcription of adipogenic genes, resulting in phenotypically and functionally different fat cells. Likewise, extracellular signal-regulated kinases (ERKs), members of the mitogen-activated protein kinases (MAP-Ks), also participate in the signaling cascades of C/EBPα and PPARγ expression [19].

As presented in Table 1, many studies demonstrated that using EO led to suppressed lipid droplet accumulation and adipogenesis in a dose-dependent manner. Yen et al. [1], Ngamdokmai et al. [18], Hwang et al. [19], Lee et al. [20], Ko et al. [21], Cheng et al. [22], and Sprenger et al. [23] investigated the effect of various EOs, such as lemon balm, peppermint, lavender, bergamot, cypress, niaouli nerolidol, geranium-rose, revensara, lemon grass, ginger, black pepper, *Artemisa annua* L., calamus, *Pinus koraiensis*, and cinnamon. These EOs were all tested in 3T3-L1 preadipocytes treated with different concentrations during the differentiation process, followed by Oil-Red O (ORO) assay to assess lipid accumulation. The outcomes of these studies exhibited that these EOs significantly inhibited lipid accumulation through the downregulation of 3T3-L1 adipocyte differentiation. Adipogenesis inhibition is due to the suppression of adipogenic transcription factors expression, mentioned above. It is concluded that these EO have anti-obesogenic and hypolipidemic potential via inhibition of PPARγ-related signaling.

For instance, the studies performed by Ngamdokmai et al. [18], Lee et al. [19], Cheng et al. [22], Sprenger et al. [23], Lai et al. [24], and Lai et al. [25], besides testing EOs per se, also isolated the major component of each and tested their action in the model of study (in vitro or in vivo). It was concluded that those components exerted anti-obesity effects.

In the case of olfactory stimulation, the study performed by Hong et al. [15] demonstrated that citronellol is a volatile and prominent patchouli EO (PEO) compound that is accountable for diminishing food intake, thus preventing obesity. Moreover, α-patchoulene and β-patchoulene release the PEO odor, stimulating the hypothalamus and regulating serum leptin levels, lowering food intake.

However, as Russo et al. [7] mentioned, further investigation is required for individual components’ action in adipocyte metabolism since EOs are phytochemically complex molecules in which each component is thought to take part in the overall outcome and could regulate the effect of the others, either synergistically or antagonistically. Therefore, it could be uncertain which individual components are the only ones responsible for conferring anti-obesity effects, thus it is preferable to assert results taking into consideration the EO as a whole.

Studies in animals have demonstrated that certain EOs exert anti-obesogenic activity. Ko et al. [21], Cheng et al. [22], Lai et al. [24], Lai et al. [25], Asnaashari et al. [26], and Ciftci et al. [27] investigated the effect of garlic, *Pinus koraiensis*, lime, cinnamon, ginger and a mix of thyme, orange peel, bay leaf, and eucalyptus, respectively, on body weight, food intake, serum biochemical metabolites (glucose, insulin, free fatty acids, cholesterol, and triglycerides), and adipose tissue of standard or high-fat diet (HFD) fed animals (mice, rats or quails). EOs tested suppressed—in a dose-dependent manner—increases in fat pads, body weight, and serum biochemical parameters induced by HFD.

Scientific reports evaluating the anti-obesity effects of EO compared to orlistat—a specific gastrointestinal lipase inhibitor commonly used in obesity treatment that inhibits the absorption of fat, resulting in weight loss—concluded that sweet orange and cumin EOs are potential candidates to replace pharmacological obesity treatments through downregulation of PPARγ expression, consequently preventing preadipocytes [28,29,30,31].

**Table 1:** Studies evaluating the effect of essential oils (EO) on the prevention and treatment of obesity.

Essential Oil (EO)	Study Details	Experimental Findings	Reference
29 different EOs (lemon balm, Spanish sage, rosemary, marjoram, peppermint, lavender, thyme, basil, orange, bergamot, lemon, mandarin, grapefruit, tea tree, Niaouli nerolidol, eucalyptus, cypress, cedarwood, juniper-berry, black pepper, frankincense, ginger, geranium-rose, fennel, chamomile-roman, pine, and even Sara)	In vitro—3T3-L1 preadipocytes were differentiated. Oil-Red O (ORO) stain assay was done to assess lipid accumulation. 3T3-L1 adipocytes were treated with all samples at 60 µL/mL concentration for six days.	Lemon balm, peppermint, lavender, bergamot, cypress, niaouli nerolidol, geranium-rose, and revensara inhibited lipid accumulation by 53–90% compared to the control. Spanish sage, rosemary, marjoram, orange, eucalyptus, cedarwood, black pepper, and ginger increased lipid accumulation (110–167%). Thyme, lemon, tea tree, fennel, chamomile-roman, pine, basil, mandarin, grapefruit, juniper-berry, and frankincense did not show effects on lipid metabolism (90–110%).	[1]
Garlic EO (GEO)	In vivo—Six-week-old male C57BL/6J mice were fed a standard or high-fat diet (HFD) with and without GEO for 12 weeks. GEO concentrations were 25, 50, or 100 mg/kg. Blood, liver, subcutaneous, epididymal, and perirenal fats were collected.	GEO at 50 mg/kg concentration prevented the increment of subcutaneous, epididymal, and perirenal fat pads in mice fed with HFD, and reduced their elevated glucose levels, insulin, free fatty acids, and triglycerides.	[24]
Seven different EOs (lemon grass, ginger, black pepper, long pepper, turmeric, cassumunar ginger, and kaffir lime)	In vitro—3T3-L1 preadipocytes were differentiated. ORO stain assay was done to assess lipid accumulation. Total triglyceride content was determined using a triglyceride assay kit.	All EO inhibited or decreased lipid accumulation, adipogenesis, and triglyceride content.	[18]
*Artemisia annua* L. EO	In vitro—3T3-L1 preadipocytes were differentiated. ORO stain assay was done to assess lipid accumulation.	Inhibited adipogenesis.	[20]
Calamus EO	In vitro—After 3T3-L1 differentiation, a total triglycerides assay, ORO, RT-PCR, and western blot analyses (for analysis of p-ERK1/2, C/EBPβ, C/EBPα, and PPARγ protein) were conducted.	Reduction of intracellular triglyceride content and adipogenesis inhibition was detected.	[19]
*Pinus koraiensis* EO (PKEO)	In vitro—ORO staining, triglycerides content, and expression levels of adipogenic factors were measured in 3T3-L1 differentiated cells treated with PKEO.	In vitro results showed a reduction of intracellular triglyceride content and downregulation of adipogenic transcription factors expression.	[21]
	In vivo—Male Sprague-Dawley rats, at four weeks of age, treated with high-fat diets, whose body weights, retroperitoneal and epididymal fats, and serum lipid metabolites (HDL, LDL, triglycerides) were assessed during six weeks.	In vivo results demonstrated that PKEO treatment prevented weight gain and suppressed serum triglyceride, total cholesterol, and LDL cholesterol.	
Lime EO	In vivo—Fifty-six male mice weighing 25–30 g were divided into seven groups for 45 days. Males were subcutaneously treated with normal saline (0.1 mL/mice), DMSO (0.02 mL/mice), ketotifen dissolved in 0.1 mL of normal saline (32 mg/kg), lime EO dissolved in 0.02 mL of DMSO (125, 250, 500 mg/kg), and a mixture of ketotifen and lime EO (32 mg/kg, and 125 mg/kg, respectively) properly dissolved in normal saline and DMSO, respectively. Food intake and body weight changes were studied.	Mice treated with lime EO exhibited both body weight loss and food intake reduction.	[26]
Cinnamon EO (CEO)	In vitro—3T3-L1 cells were differentiated with the EO, and their major components, S-(+)-linalool, and R-(-)-linalool. After differentiation, the ORO assay was performed.	In vitro results exhibited that treatment with cinnamon EO reduced the accumulation of lipid droplets, S-(+)-linalool, and R-(-)-linalool compared with the control group. Higher doses (100 µg/mL) improved the inhibition effect more than lower ones (10 µg/mL).	[22]
	In vivo—Six-week-old male ICR mice were orally treated with corn oil as control, 250 and 500 mg/kg of CEO, 500 mg/kg of S-(+)-linalool, and 500 mg/kg of R-(-)-linalool, for 14 days. Body weight changes and blood biochemical parameters (glucose, total cholesterol (TC), triglyceride levels (TG)) were monitored.	In vivo results demonstrated that the body weight change rate was lower than the control group for those mice treated with CEO and S-(+)-linalool. As well as this, blood glucose, TC, and TG were decreased.	
Citronella EO	Clinical trial—A randomized, double-blind, placebo-controlled clinical trial was conducted with 78 overweight subjects aged between 18 and 60. Participants were divided into three groups: (1) treated with 100 mg EO of *Cumin cyminum* L. capsule; (2) treated with orlistat120 capsule, and (3) treated with placebo. Treatments were taken three times per day for eight weeks. Anthropometric measures and fasting blood samples were taken at baseline and after treatments.	Participants who were treated with EO of *Cumin cyminum* L. capsule exhibited a decrease in weight and body mass index compared to orlistat120 and placebo. Likewise, cumin EO capsules reduced serum insulin levels.	[28]
Ginger EO (GgEO)	In vivo—Eight-week-old male C57BL/6J mice were fed a standard diet or HFD for 12 weeks with orally administrated GgEO or citral (its main chemical compound). They were divided into four groups: (1) positive control with a standard diet with 13.5% kcal fat content; (2) negative control with an HFD with 60% kcal fat content; (3) HFD + GEO (12.5, 62.5, or 125 mg/kg) and (4) HFD + citral (2.5 or 25 mg/kg). Food intake and body weight were monitored. Serum biochemical parameters (glucose, insulin, free fatty acids, cholesterol, and triglycerides) were assessed. Liver, subcutaneous, epididymal, and perirenal adipose tissue were collected.	GgEO and citral treatments reduced average body weight by preventing the HFD-treated mice increasing their amount of subcutaneous, epididymal, and perirenal fat pads in a dose-dependent manner. These same treatments considerably decreased the results of serum biochemical levels in a dose-dependent manner.	[25]
Grapefruit EO (GpEO)	In vivo—Male Wistar rats (250–300 g) and male C57BL/6J mice were subjected to olfactory stimulation with GpEO. Autonomic nerve activities were examined electro-physiologically by placing the nose of the anesthetized rat inside a beaker that contained filter paper soaked in GpEO or water. To assess the effects of GpEO on food intake and body and tissue weights, a gauze soaked in GpEO was placed above the animal cage for 15 min, three times a week, for six weeks.	Sympathetic white and brown adipose tissue nerve was increased with GpEO inhalation treatment. GpEO reduced food intake, body weight, and organs and adipose tissue weights.	[31]
Patchouli EO (PEO)	In vivo—Four-week-old male Sprague Dawley rats were divided into four groups: (1) standard diet fed control + 30-min inhalation of distilled water (DW); (2) HFD fed control + 30-min inhalation of DW; (3) and (4) HFD + 0.3% and 1% PEO 30-min inhalation, respectively. All treatments lasted 12 weeks. Body weight, food intake, and serum biochemical parameters (TC, HDL cholesterol, and TG) were measured for all groups. Brain, heart, kidney, liver, white adipose tissue (WAT), and brown adipose tissue (BAT) were extracted.	Groups subjected to PEO inhalation treatments exhibited a decrement in food intake and body weight. BAT weight was decreased. HDL cholesterol was increased while LDL was decreased.	[15]
Sweet orange EO (SOEO)	In vivo—Four- to six-week-old male Sprague Dawley rats (190–210 g) were divided into six groups: (1) HFD + 2 mL of normal saline; (2) HFD + 2 mL of β-cyclodextrin; (3) HFD + 19 mg of SOEO + 2 mL of normal saline; (4) HFD + 2 mL suspension of SOEO microcapsules (microcapsules were made with SOEO + β-cyclodextrin); (5) HFD + 2 mL suspension of orlistat powder and (6) rats treated with a low-fat diet. Rats were subjected to treatments for 15 days. Body weight and food intake were assessed every two days. Serum biochemical analysis was done.	SOEO microcapsules significantly lowered body weight gain and fat rate compared to HFD-fed rats. Furthermore, SOEO microcapsules decreased total cholesterol and LDL cholesterol levels in serum.	[29]
Lemongrass EO (LGEO)	In vitro—ORO staining, triglycerides content, and expression levels of adipogenic factors were measured in 3T3-L1 differentiated cells treated with LGEO and its major constituents: citral and citral diethyl acetal.	LGEO and its major constituents decreased lipid accumulation via adipogenesis inhibition, increased lipolysis, and decreased lipid uptake.	[23]
Mix of EO (MEO) composed of thyme (50%), orange peel (25%), bay leaf (12.5%), and eucalyptus (12.5%) EO	In vivo—15-day-old Japanese quails were divided into three groups and exposed to a low ambient temperature. Treatments were: (1) basal-diet; (2) basal diet + 50 ppm of MEO; and (3) basal-diet + 100 ppm of MEO. Serum biochemical parameters were measured.	MEO decreased serum glucose, TG, and TC compared to the control group.	[27]

### 3.2. Antidiabetic Potential

Diabetes mellitus (DM) is a chronic, lifelong progressive metabolic disorder caused by impaired insulin secretion or insulin resistance, resulting in chronic hyperglycemia. Metabolic abnormalities in carbohydrates, lipids, and proteins arise as a result of low levels of insulin to achieve adequate response or insulin resistance in target tissues [32,33,34].

Two primary factors are involved in the development of type 2 diabetes (T2D): impaired insulin secretion by pancreatic β-cells or a lowered number of β-cell mass, which may also contribute to insufficient secretion of insulin and the inability of insulin-sensitive tissues to respond appropriately to this hormone [35]. Insulin resistance in T2D increases the demand for insulin in insulin-target tissues. However, this increased demand for insulin could not be met by the pancreatic β cells due to defects in the function of these cells, which in turn decreases insulin secretion due to the gradual destruction of β cells, resulting in a vicious cycle of metabolic state worsening that could transform some type 2 diabetes patients from being independent to becoming dependent on insulin [33].

#### 3.2.1. Postprandial Hyperglycemia

Postprandial hyperglycemia has been described in the etiology of T2D and cardiovascular disease (CVD); moreover, it is a significant risk factor for the development of atherosclerosis in nondiabetic people [36,37]. Postprandial hyperglycemia is an excessive plasma glucose concentration after eating, characterized by hyperglycemic spikes that induce oxidative stress. Even in healthy subjects, short-term postprandial hyperglycemia is accompanied by endothelial dysfunction, elevated adhesion molecules, and proinflammatory cytokines in the blood. Postprandial hyperglycemia is driven by many factors such as timing, quantity, meal composition, carbohydrate content, insulin and glucagon secretion, among others [37,38,39]. Regarding adipocytes, they are the principal targets for postprandial glucose uptake [1].

#### 3.2.2. Starch and Digestive Enzymes Activity

Carbohydrates are the main dietary component that affects glycemia [36]. Once a meal rich in carbohydrates is ingested, starch is hydrolyzed quickly by digestive enzymes such as α-amylase and α-glucosidase, which results in a high rise in blood glucose and insulin level [40].

Starch contributes to 40–60% of the total energy intake in the human diet [41]. This complex carbohydrate comprises two glucose polymers: amylose, a linear polymer composed of glucose units linked by alpha-(1→4) bonds, and amylopectin, which is a large branched molecule that also has glucose chains linked by alpha-(1→4) bonds and also has glucose chain branches with alpha-(1→6) bonds [42].

In humans, α-amylases are found in the salivary glands that secrete the enzyme in the mouth and the pancreas, which secretes the enzyme in the small intestine. It hydrolyzes the α-(1→4) glycosidic bonds in the starch molecule leading to the production of maltose, maltotriose, maltotetraose, maltodextrins, and glucose [42].

For its part, α-glucosidase is found on the luminal surface of enterocytes and is secreted in the small intestine. It is a key enzyme that catalyzes the hydrolysis of disaccharides (maltose and sucrose) into monosaccharides (glucose and fructose) and acts predominantly on α-amylase digestion products, rapidly converting them to glucose. Likewise, α-glucosidase can hydrolyze α-(1→6) bonds, which cannot be attacked by α-amylase, removing dextrins and allowing starch digestion to complete [41].

#### 3.2.3. Diabetes Pharmacological Therapy

Diabetes management includes glycemic control, reducing body weight, changes in lifestyle, prevention of micro and macrovascular damage, and others. Glycemia in type 2 diabetes patients can be controlled by pharmacological therapy. Four main groups of antidiabetic drugs act through different mechanisms: (i) biguanides: reduce gluconeogenesis in the liver (e.g., metformin); (ii) insulin secretagogues: stimulate insulin secretion of the pancreas (e.g., sulfonylureas); (iii) insulin sensitizers: improve the sensitivity of peripheral tissues to insulin (e.g., thiazolidinediones); and (iv) insulin or its analogs which provide insulin exogenously in the form of recombinant insulin [43].

In the case of thiazolidinediones, they act via the activation of peroxisome proliferator-activated receptors (PPARs), decreasing insulin resistance and regulating adipocyte differentiation. For instance, biguanides, such as metformin, act by activating adenosine monophosphate-activated protein kinase (AMPK), which plays a significant role in energetic balance, insulin signaling, and metabolism of fats and glucose [1]. Additionally, metformin affects the translocation of GLUT4 in insulin-targeted cells. GLUT4 is an ATP-independent glucose transport protein prevalent in adipose and muscle tissues and enhances glucose uptake. Metformin also activates AMPK phosphorylation in adipose and muscle tissues; this mechanism compromises phosphorylation of insulin receptor substrate 1 (IRS-1) Ser789, which, via cascade signaling, activates phosphoinositide 3 kinase/protein kinase B (PI3K/PKB) signaling, thus increasing blood glucose balance and decreasing insulin resistance. In adipocytes, AMPK activation inhibits lipogenesis while enhancing energy consumption, leading to an anti-obesity effect [44]. Both AMPK activators and PPARs ligands regulate glucose homeostasis and decrease insulin resistance in adipose tissue [1].

Control of postprandial hyperglycemia is an essential factor in diabetes treatment. Currently, there are three main oral antidiabetic drugs: acarbose, miglitol, and voglibose, which regulate glucose availability for intestinal absorption by modifying carbohydrate digestion. All of these drugs are 𝛼-glucosidase inhibitors that reversibly and competitively reduce the hydrolytic activity of these enzymes, thereby regulating the availability of glucose for intestinal absorption and the speed and extent of postprandial hyperglycemia [45].

Acarbose has been used as a pharmacological prescription to manage postprandial glucose. It has been reported that it can decrease diabetes progression by 25% [36]. Drug combination therapeutic management has shown better results than drug monotherapy; therefore, acarbose and metformin treatment has been reported to improve effects on patients with T2D [43]. However, acarbose has common gastrointestinal adverse effects, including abdominal pain, diarrhea, and bloating [46]. These side effects result from maltose fermentation, accumulating due to α-glucosidase inhibition [47]. The difference in the mechanism of action of acarbose to miglitol and voglibose is that the former reduces polysaccharides digestion in the upper small intestine. In contrast, the latter reduces disaccharide digestion, thus in the lower small intestine there is a higher polysaccharide content when consuming acarbose; with miglitol and voglibose, there is a higher disaccharide content in the lower small intestine [45].

Postprandial hyperglycemia in nondiabetic people is a predictor of insulin resistance and cardiovascular disease. In the case of patients with T2D, it has a relationship with micro and macrovascular disease. Moreover, sharp long-term changes in blood insulin levels in normal individuals may cause insulin resistance in organs and tissues, a central mark of hyperglycemia and type 2 diabetes. Regulating postprandial hyperglycemia early is a feasible strategy for preventing and managing T2D [40].

#### 3.2.4. EO as an Alternative to Pharmaceutical Drugs

Hence, common pharmaceutical approaches in the management and treatment of T2D compromise AMPK activators, PPARs ligands, and α-amylase and α-glucosidase inhibitors, which moderate the metabolism of dietary carbohydrates [48]. Nevertheless, undesirable effects are displayed by these treatments, which could be attenuated by EOs exerting antidiabetic effects (Table 2).

In the case of carbohydrate-related enzymes, which regulate carbohydrate digestion and glucose absorption in the small intestine, it has been reported that partial inhibition of α-amylase and α-glucosidase by EO is a natural alternative in the control of T2D. In the study performed by Radünz et al. [49], among all the EOs evaluated, thyme offered the most significant α-glucosidase inhibition (98.9%), while sweet orange EO showed the most potently inhibitory effect in α-amylase (95.4%). Their major components, thymol and D-limonene, respectively, are thought to be responsible for inhibitory capacity. All EO evaluated in this study exhibited a better capacity for enzyme inhibition than acarbose, the conventional drug prescribed. Moreover, incomplete enzyme inhibition, and medium and high range inhibition for α-amylase and α-glucosidase, respectively, is proposed for clinical treatment since these ranges allow the control of T2D without compromising nutrients or glucose absorption in the small intestine.

According to Rahali et al. [50], some important factors need to be considered respecting the biological activity of plant EO. Even though its chemical composition is responsible for conferring bioactivities, it is influenced by plant genotype, organ type, extraction type, phenological stage, and environmental conditions. Their study analyzed the chemical composition of different plant organs, such as leaves, flower buds, flowers, and fruits, in terms of the EO of *Hertia cheirifolia*, and how these differences influenced α-amylase inhibitory activities. It was reported that leaves and fruits EO possessed the highest activity of α-amylase inhibition with 8.32 and 8.84 mg Eq acarbose/g EO, respectively.

In this regard, the study performed by Pavlić et al. [51] evaluated different extraction techniques and experimental conditions in peppermint leaves. Extraction methods included conventional hydrodistillation (HD), microwave-assisted hydrodistillation (MWHD), soxhlet extraction (SOX), ultrasound-assisted extraction (UAE), microwave-assisted extraction (MAE), and supercritical fluid extraction (SFE). HD and MWHD were applied to obtain the volatile fraction, that is, pure EO. It is expected that polyphenols and flavonoids were not present in these samples. The rest of the techniques aimed to recover lipophilic compounds, which are mixtures of volatile and non-volatile lipids. Its chemical composition varied depending on the extraction method since some monoterpene hydrocarbons (α-pinene, camphene, myrcene, and terpinolene) were absent in SOX, MAE, and UAE. For instance, SFE allows the extraction of terpenoids (oxygenated compounds) and other lipophilic bioactive compounds. Results of this study showed that peppermint EO, obtained by HD and MWHD, was the most potent α-amylase inhibitor, with an activity range of 1.24–1.76 ACEs/g. However, EOs did not exhibit α-glucosidase inhibition, while most lipophilic extracts were potent inhibitors with a 57.96–58.89 mmol ACEs/g activity range.

Studies in animals have demonstrated that lemon balm EO has antihyperglycemic effects. The study carried out by Chung et al. [52] demonstrated that supplementation to mice fed with lemon balm EO showed a decrease in glucose concentration and an increment in glucose tolerance. These results indicated that lemon balm EO stimulates glucokinase (GCK) activity and inhibits glucose-6-phosphatase (G6Pase) activity in the liver of mice. The former is stimulated by insulin and enhances glucose consumption and uptake in the liver, while the latter controls hepatic gluconeogenesis and glucose output in the liver; it is inhibited by insulin. When its activity is reduced, it decreases hepatic glucose production.

As presented in Table 2, some EO exhibited a lower carbohydrate-enzymatic-related inhibition activity when using acarbose as a positive control [53,54,55,56,57]. However, the side effects of synthetic drugs used to treat obesity and diabetes are not expected to happen with natural compounds like EO.

**Table 2:** Studies evaluating the effect of essential oils (EO) on the prevention and treatment of diabetes.

Essential Oil (EO)	Study Details	Experimental Findings	Reference
Clove, thyme, oregano, and sweet orange	Enzymatic assay—EO were extracted by hydrodistillation for 3 h using a Clevenger apparatus. α-amylase and α-glucosidase inhibition colorimetric assays were assessed. Experimental concentrations for each EO were 250 mg/mL. Acarbose was used as a positive control.	α-amylase inhibition Clove, thyme, oregano, sweet orange EO, and acarbose inhibited 93.1, 81.3, 81.4, 95.4, and 73.5% of α-amylase activity, respectively. α-glucosidase inhibition Clove, thyme, oregano, sweet orange EO, and acarbose inhibited 75.5, 98.9, 50.5, 37.3, and 34.5% of α-glucosidase activity, respectively.	[49]
Wild mint *(Mentha longifolia* var. *calliantha*)	Enzymatic assay—EO was extracted by hydrodistillation for 3 h using a Clevenger apparatus. α-amylase and α-glucosidase inhibition assays were assessed using 3,5-dinitrosalisylic acid (DNS) and p-nitrophenyl-α-D-glucopyranoside (pNPG) methods, respectively. Enzymes’ inhibitory activity was expressed as equivalents of acarbose (ACEs).	α-amylase inhibitory activity: 2.74 mmol ACEs/g EO α-glucosidase inhibitory activity: 5.62 mmol ACEs/g EO	[58]
*Hertia cheirifolia*	Enzymatic assay—EO from leaves, flower buds, flowers, and fruits were extracted by hydrodistillation for 3 h using a Clevenger apparatus. α-amylase inhibition assay was assessed using the DNS method. Results were expressed as equivalent acarbose per gram of EO (ACEs).	α-amylase inhibitory activities in different plant organs: Leaves: 8.32 mg ACEs/g EO Flower buds: 2.75 mg ACEs/g EO Flowers: 5.85 mg ACEs/g EO Fruits: 8.84 mg ACEs/g EO	[50]
*Nepeta curviflora*	Enzymatic assay—EO was extracted utilizing a microwave ultrasonic apparatus. α-amylase and α-glucosidase inhibition assays were assessed using DNS and pNPG, respectively. Experimental concentrations for α-amylase assay were 10, 50, 70, 100, and 500 μg/mL, while for α-glucosidase they were 100, 200, 300, 400, and 500 μg/mL.	The highest inhibitory percentage for α-amylase was 65.8%, achieved with a concentration of 500 μg/mL. However, at the same concentration, acarbose presented a higher inhibitory activity (72.54%). Nepeta curviflora EO IC50 in this assay was 45.7 μg/mL. In comparison, acarbose IC50 was 28.84 μg/mL. In the case of α-glucosidase, the highest inhibitory percentage was 92.72% with a concentration of 500 μg/mL. It had a slightly higher inhibitory activity than acarbose at the same concentration (92.28%). Nepeta curviflora EO IC50 in this assay was 54.9 μg/mL. In comparison, acarbose IC50 was 37.15 μg/mL.	[53]
*Oliveria decumbens* (OD), *Thymus kotschyanus* (TK), *Trachyspermum ammi* (TA), and *Zataria multiflora* (ZM) EO	Enzymatic assay—EOs were extracted by hydrodistillation for 3 h using a Clevenger apparatus. α-amylase and α-glucosidase inhibition colorimetric assays were assessed.	α-amylase inhibition OD IC50: 223 μg/mL TK IC50: 229 μg/mL TA IC50: 218 μg/mL ZM IC50: 216 μg/mL Acarbose IC50: 126 μg/mL α-glucosidase inhibition OD IC50: 220 μg/mL TK IC50: 238 μg/mL TA IC50: 212 μg/mL ZM IC50: 219 μg/mL Acarbose IC50: 139 μg/mL For both assays, all EOs similarly inhibited enzymes but at lower levels than acarbose.	[54]
*Cedrus libani*	Enzymatic assay—EO from wood, leaves, and cones was extracted by hydrodistillation for 3 h using a Clevenger apparatus. α-amylase inhibition colorimetric assay (DNS) was performed. Experimental concentrations range from 1 mg/mL to 0.1 mg/mL.	Wood EO IC50: 0.14 mg/mL Cone EO IC50: >1 mg/mL Leaves did not demonstrate inhibition.	[59]
Orange and lemon peels EO	Enzymatic assay—EOs were extracted by hydrodistillation for 3 h using a Clevenger apparatus. α-amylase and α-glucosidase inhibition colorimetric assays were assessed. Experimental concentrations for each EO were 0–16 μg/mL.	α-amylase inhibition Orange peel IC50: 11.51 μg/mL Lemon peel IC50: 8.16 μg/mL Acarbose IC50: 7.45 μg/mL α-glucosidase inhibition Orange peel IC50: 11.53 μg/mL Lemon peel IC50: 7.56 μg/mL Acarbose IC50: 8.44 μg/mL Lemon peel EO exhibited the highest inhibitory effects in both enzymes. The α-glucosidase inhibition assay has a higher inhibitory effect than acarbose.	[55]
Black pepper	Enzymatic assay—EO was extracted by hydrodistillation for 3 h using a Clevenger apparatus. α-amylase and α-glucosidase inhibition colorimetric assays were performed. Experimental concentrations for each EO were 0–120 mL/L.	α-amylase inhibition IC_50_: 86.06 mL/L α-glucosidase inhibition IC_50_: 68.29 mL/L Black pepper EO showed more potent inhibitory activity in α-glucosidase than in α-amylase.	[60]
Peppermint (*Mentha piperita* L.)	Enzymatic assay—different extraction methods for EO: conventional hydrodistillation (HD); microwave-assisted hydrodistillation (MWHD); soxhlet extraction (SOX); ultrasound-assisted extraction (UAE); microwave-assisted extraction (MAE); and supercritical fluid extraction (SFE). α-amylase and α-glucosidase inhibition colorimetric assays were assessed.	α-amylase inhibitory activity range: 1.24–1.76 mmol ACEs/g α-glucosidase inhibitory activity range: 57.96–58.89 mmol ACEs/g	[51]
Lavender	In vivo—15-weeks-old adult male Wistar rats (220–230 g) were divided into four groups: (1) control (nondiabetic rats) treated with 0.9% NaCl; (2) alloxan-induced diabetic rats treated with 0.9% NaCl; (3) nondiabetic rats treated with EO (50 mg/kg body weight); and (4) alloxan-induced diabetic rats treated with EO (50 mg/kg body weight). Treatments lasted 15 days. Serum biochemical parameters were determined. EO was extracted by hydrodistillation for 3 h using a Clevenger apparatus.	There was a significant increase in blood glucose levels within alloxan-induced diabetic rat groups; however, treatment with EO significantly reduced this parameter.	[61]
*Origanum vulgare* subsp. *vulgare* and subsp. *hirtum*	Enzymatic assay—EO were extracted by hydrodistillation for 5 h using a Clevenger apparatus. α-amylase and α-glucosidase inhibition colorimetric assays were performed.	For α-amylase inhibitory activity, *Origanum vulgare* subsp. *vulgare* and subsp. *hirtum* exhibited similar activity (0.13 and 0.14 mmol ACEs/g oil). The highest α-glucosidase inhibitory activity was achieved by *Origanum vulgare* subsp. *vulgare* with 6.04 mmol ACEs/g oil.	[62]
Clove bud	Enzymatic assay—EO was extracted by hydrodistillation for 3 h using a Clevenger apparatus. α-amylase and α-glucosidase inhibition colorimetric assays were performed. Experimental concentrations were 0, 40, 80, 120, and 160 µL/L. Acarbose was used as a positive control.	35–78% inhibition of α-amylase. 58–90% inhibition of α-glucosidase. Clove bud oil EC_50_ for α-amylase: 88.89 µL/L Clove bud oil EC_50_ for α-glucosidase: 71.94 µL/L Acarbose EC_50_ for α-amylase: 18.63 µg/mL Acarbose EC_50_ for α-amylase: 21.1 µg/mL Acarbose exhibited higher inhibitory activity for both enzymes compared to clove bud EO.	[56]
*Cinnamomum zeylanicum* (CZ), *Psiadia arguta* (PA), *Psiadia terebinthina* (PT), *Citrus grandis* (CGp), *Citrus hystrix* (CH), and *Citrus reticulata* (CR)	Enzymatic assay—EO were extracted by hydrodistillation for 3 h using a Clevenger apparatus. α-glucosidase inhibition assay was assessed using the pNPG method. The inhibition type was determined using the Lineweaver-Burk linearization method.	Inhibition % at 500 µg/mL for CH, CR, CGp, CZ, PT, and PA was 85.49, 81.15, 83.19, 93.71, 40.12, and 76.45, respectively. IC50 (µg/mL) values are 276.7, 169.9, 240.6, 64.52, 14,584, and 313, respectively. In the case of inhibition %, all EO exhibited higher activity than acarbose (51.39%). CZ was demonstrated to be the most potent inhibitory activity compared to acarbose. For all EOs, the inhibition type was uncompetitive, except for CZ, which has a competitive inhibition type.	[63]
Lemon balm (*Melissa officinalis*)	In vivo—15-weeks-old male C57BL/KsJ-db/db (db/db) mice were fed with standard chow or chow supplemented with lemon balm EO. Treatments lasted for six weeks. Serum biochemical parameters were monitored. Oral glucose tolerance tests were assessed, and serum insulin was monitored. EO was extracted by steam distillation.	Plasma glucose levels were reduced (up to 64.6%). There was an improvement in glucose tolerance with lemon balm EO administration. Serum insulin was increased. Serum biochemical parameters (total cholesterol, TG and HDL-cholesterol) were reduced.	[52]
*Phoebe bournei* (Hemsl.) Yang	In vitro—3T3-L1 preadipocytes were differentiated with 40 µg/mL of leaf EO. After 24 h, glucose consumption activity was determined by measuring the medium glucose concentration.	Promotion of glucose uptake in adipocytes.	[64]
Rosemary	In vivo—15-weeks-old adult male Wistar rats (220–225 g) were divided into four groups: (1) nondiabetic rats treated with distilled water; (2) alloxan-induced diabetic rats treated with distilled water; (3) nondiabetic rats treated with EO; and (4) alloxan-induced diabetic rats treated with EO. Treatments lasted 15 days. Blood glucose level was measured. EO was extracted by hydrodistillation for 3 h using a Clevenger apparatus.	Blood glucose level was higher in alloxan-induced diabetic rats; however, treatments with EO corrected this hyperglycemia.	[65]
*Rhaponticum acaule* (L) DC	Enzymatic assay—EO was extracted by hydrodistillation for 5 h using a Clevenger apparatus. α-glucosidase inhibition assay was assessed using the pNPG method. The inhibition type was determined using the Lineweaver-Burk method.	*Rhaponticum acaule* EO IC50: 6.7 ± 0.10 μg/mL Acarbose IC50: 280 ± 0.10 μg/mL EO demonstrated high inhibition activity compared to acarbose. Mixed inhibition type.	[66]
*Salvia officinalis* L.	EO was extracted by hydrodistillation for 2 h using a Clevenger apparatus. Enzymatic assay—α-amylase inhibition assay was assessed using the CNPG3 method. Experimental concentrations were 50, 100, and 200 µg/mL. In vivo—Male Wistar rats (180–200 g) were induced into diabetes with alloxan and divided into five groups: (1) nondiabetic rats treated with water (control); (2) nondiabetic rats treated with EO; (3) alloxan-induced diabetic rats treated with water; (4) alloxan-induced diabetic rats treated with Glymepiride; and (5) alloxan-induced diabetic rats treated with EO. Fasting blood glucose, α-amylase, and hepatic glycogen content were measured. Treatments were daily and orally administered.	EO IC_50_: 38 μg/mL Acarbose IC_50_: 14.9 μg/mL EO exhibited less inhibition activity than acarbose. EO administration to diabetic rats reduced serum α-amylase activity and fasting blood glucose. Moreover, liver glycogen storage was enhanced by 44%. Langerhans islets were restored to normal size in diabetic rats.	[57]

### 3.3. Other Bioactivities of EO Related to Metabolic Syndrome Comorbidities: Neuroprotection

Diabetes is a risk factor for developing Alzheimer’s disease (AD) and other types of dementia [67]. In this context, untreated diabetes can cause memory disorders [68]. Because of chemical properties or monoterpenes, they can travel quickly across the single epithelial nasal mucosa, be incorporated into blood circulation, and cross the blood-brain barrier. For those reasons, aromatherapy with EO has been an alternative to AD treatment [67].

Furthermore, cholinesterase inhibitors are the target for preventing and treating AD. These inhibitors impede the cholinergic deficit associated with cognitive dysfunction [63]. Two principal cholinesterases, acetylcholinesterase (AChE) and butyrylcholinesterase (BChE), are associated with AD [58]. An increase in these cholinesterases leads to reduced levels of acetylcholine neurotransmitter, which is involved in memory and learning [67]. For instance, AChE is related to β-amyloid plaques and neurofibrillary tangles (NFT) [69]. This inhibition promotes an increase in the level of acetylcholine in neuronal synapsis, which leads to improved stimulation of the cholinergic receptors [62,63].

Wild mint EO has been evaluated as a potential cholinesterase inhibitor. The study performed by Asghari et al. [58] showed that wild mint (*Mentha longifolia* var. *calliantha*) EO has an intense AChE activity. Enzymes’ inhibitory activity was expressed as equivalents of galantamine (GALAEs). In the case of AChE, IC50 was 1.82 mg GALAEs, and 2.57 mg GALAEs for BChE. This study mentioned that oxygenated monoterpenes present in the EO were accountable for neuroprotection since 1,8-cineol, the most abundant component, and carvacrol have been reported to be acetylcholinesterase inhibitors.

The study by Sarikurkcu et al. [62] suggested that *Origanum* species are recommended for AD treatment. It was reported that *O. vulgare* subsp. *vulgare* and *O. vulgare* subsp. *hirtum* exhibited a similar inhibitory action on both AChE and BChE. It is thought that inhibitory properties are due to the high concentration of thymol and carvacrol in *O. vulgare* subsp. *vulgare*, while for *O. vulgare* subsp. *hirtum* it is because of the high concentration of linalool.

Another study conducted by Aumeeruddy-Elalfi et al. [63] proposed that citrus species such as *Citrus grandis* (CGp), *Citrus hystrix* (CH), and *Citrus reticulata* (CR) have comparable activity as galantamine, a common drug used to treat mild to moderate AD. These EO are categorized as an uncompetitive type of inhibitor since there is a decrement in K_m_ and V_max_ parameters in their presence. Discovering the inhibition type would be helpful for further investigations to achieve, so as to elucidate the interaction of the EO with cholinesterases.

## 4. Bioavailability and Mechanisms of Action of EO

EOs are a complex mixture of volatile and non-volatile compounds, such as hydrocarbons, fatty acids, sterols, carotenoids, waxes, and flavonoids. In the case of volatile constituents, there are hydrocarbons (e.g., pinene, limonene, bisabolene), alcohols (e.g., linanol), acids (e.g., benzoic acid), aldehydes (e.g., citral), cyclic aldehydes (e.g., cuminal), ketones (e.g., camphor), lactones (e.g., bergaptene), phenols (e.g., eugenol), phenolic ethers (e.g., anethole), oxides (e.g., 1,8 -cineole), and esters (e.g., geranyl acetate) [70,71].

The biological properties of EO are commonly attributed to the main molecules at the highest concentrations. However, it is thought there is a synergistic relationship between the molecules in the EO. Hence, the other minor molecules could regulate the activity of major components [5]. It has been suggested that crude plant extract administration of EO is better for their bioavailability than purified compounds [44].

Investigation about the absorption, distribution, and metabolism of EO is necessary to extrapolate in vitro to in vivo studies results since therapeutic activities depend on the availability of EO compounds reaching specific target organs [72]. Some studies about the bioavailability and pharmacokinetics of monoterpenes present in EO have been published recently. For instance, oral administration of thymol and carvacrol, which are monoterpene aglycones, have a slow absorption in the bloodstream. Monoterpene aglycones have a nonpolar ending that signifies they can easily travel across cell membranes; nevertheless, their hydrophobicity is challenging. Therefore, future studies are needed to understand these compounds’ receptor interaction, activity, and specificity to elucidate their therapeutic potential [44,68].

**Figure 1:**
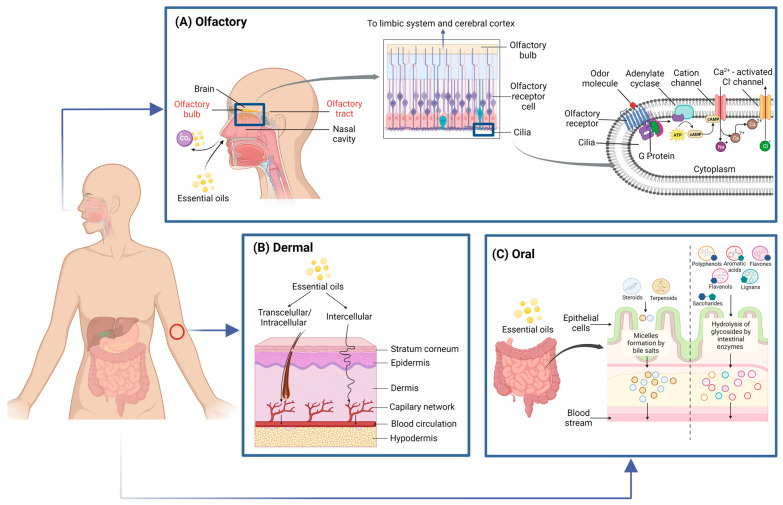
Essential oils (EO) absorption mechanisms. (**A**) Olfactory administration. Odor compounds bind to cilia in olfactory receptor cells, which activates G protein-coupled receptors to depolarize cell membranes and promote signal transduction to the limbic system and cerebral cortex. (**B**) Dermal administration. EO can penetrate the stratum corneum and reach the bloodstream through transcellular or intercellular permeation. (**C**) Oral administration. Due to their lipophilic character, terpenoids and steroids can be easily absorbed in the small intestine through lipid metabolism. Saccharide-conjugated molecules must be hydrolyzed and then metabolized in the small intestine.

It has been reported that EO can be easily absorbed via pulmonary, dermal, or oral administration (Figure 1). Regarding the pulmonary mechanism of action, smells stimulate the olfactory bulb, a part of the limbic system involved in behavioral and emotional responses, which comprises the hippocampus, amygdala, and hypothalamus. When an aromatic compound binds to cilia in olfactory receptor cells, they activate adenylate cyclase, promoting the increment in cAMP concentration. This second messenger binds to Ca^2+^ channels, causing the entrance of Ca^2+^ into the cell and depolarizing the cell membrane. Moreover, intracellular Ca^2+^ activates Cl^−^ channels and causes further membrane depolarization. These signals produce action potentials that are transferred to the glomeruli in the olfactory bulb, which eventually will be transmitted to the limbic system and cerebral cortex [70,73].

The hypothalamus oversees the autonomic nervous, endocrine, and immune systems. For instance, the autonomic nervous system (ANS) has three different divisions in terms of anatomy: sympathetic (SNS); parasympathetic (PNS); and enteric (ENS) nervous systems. In this way, studies in rats have demonstrated that some fragrances enhance sympathetic nervous activity and suppress parasympathetic activity, while others have the opposite effect on the ANS [70,74].

Olfactory stimulation studies done by Hong et al. [15], Batubara et al. [30], and Shen et al. [31] proved that citronella, grapefruit, and patchouli EO reduce body weight, as well as food intake, appetite, and plasma biochemical parameters (glucose, cholesterol, triglycerides), via sympathetic nerve stimulation in brown adipose tissue, since it promotes thermogenesis (heat production) that converts fatty acids into fuel (energy consumption). This heat generation has been reported to reduce body fat since there is an increment in mitochondria respiration and fatty acid oxidation related to AMPK activation or adipogenesis inhibition. Also, heat production can be enhanced through uncoupling protein 1 (UCP1) activation; consequently, energy consumption and body temperature are raised by uncoupling oxidation from ATP production in mitochondria. In the case of grapefruit EO, in addition to what was previously mentioned, it was also reported that it suppresses parasympathetic gastric nerve activity; as a result, it inhibits nutrient digestion and absorption.

Pulmonary absorption depends on subjects’ breathing mechanisms, mucosal compound deposition, metabolism, and the type of compound evaluated. Elimination of EO occurs mainly exhaled as CO_2_ [71,75]. Regarding dermal absorption, EO can easily be absorbed due to their lipophilic character and can easily penetrate through the skin into the bloodstream. For instance, the absorption rate in cells increases with their hydrophobicity and decreases as their molecular weight increases. Some studies have reported that after dermal administration of linalyl acetate, terpinen-4-ol, citronellol, and α-pinene, they reached their highest level 15–20 min after application and decreased gradually for 2 h [70,71,75,76,77].

The stratum corneum (SC) is the top layer of the epidermis and is a barrier to the penetration of substances. SC mainly consisted of lipids and protein keratin. EOs have been used as penetration enhancers (PE) in transdermal drug delivery systems. The role of PE is to temporarily provoke a reversible reduction in the barrier function of SC in order to allow safe and effective drug delivery via skin. EO as PE can achieve this through different mechanisms such as (i) intracellular lipid structure rearrangement between corneocytes in SC, (ii) intracellular proteins conformational modifications due to interactions, (iii) enhance drug partitioning into SC, and (iv) enhancement of desmosome connections between corneocytes or metabolic activity alteration within the skin. In this way, EOs and their active constituents can penetrate the epidermis by two different pathways: (1) transcellular (intracellular) permeation across the corneocytes of SC by appendage penetration through hair follicle, sebaceous and sweat glands; and (2) intercellular permeation through intercellular spaces of the SC. Briefly, a drug has to travel across continuous layers of intracellular lipids and proteins to reach blood circulation via the skin [77,78,79].

When orally administered, EOs interact with digested food. The kinetic rate depends on digestive enzymes to hydrolyze EO compounds from the fatty acid linkages. In the case of terpenoids and steroids present in EO, they can be digested in the small intestine along with other lipids due to their lipophilic properties. On the other hand, hydrophilic EO compounds, such as polyphenols, flavones, flavanols, lignans, and aromatic acids, are bound to saccharides metabolized in the small intestine. Aglycones that are not absorbed cross to the liver, where they are absorbed and enzymatically degraded. Free hydrophilic molecules are transported into enterocytes via passive diffusion or active transport in the duodenum [71].

Intravenous administration suggests that the elimination half-life of EO in humans is about one hour. However, it has been reported that the highest concentration of active compounds from EO is two hours after administration, and after five hours, the substances have already been effectively eliminated from the bloodstream. Evidence shows that the half-life of carvacrol, thymol, eugenol, and trans-cinnamaldehyde was between 1.84 and 2.05 h. Elimination of EO occurs mainly by renal secretion in the form of glucuronides or exhaled as CO_2_. EO are non-toxic molecules since they are fast and quickly metabolized; thus, they are not accumulated in the organism and are excreted from the body with urine and feces [71,75,77,78]. 

## 5. EO as Food Preservatives

EO have been cataloged as Generally Recognized as Safe (GRAS) for food additives and flavorings. Some EO that are GRAS include basil, cinnamon, clove, coriander, ginger, lavandin, menthol, nutmeg, oregano, rose, sage, and thyme [75].

**Figure 2:**
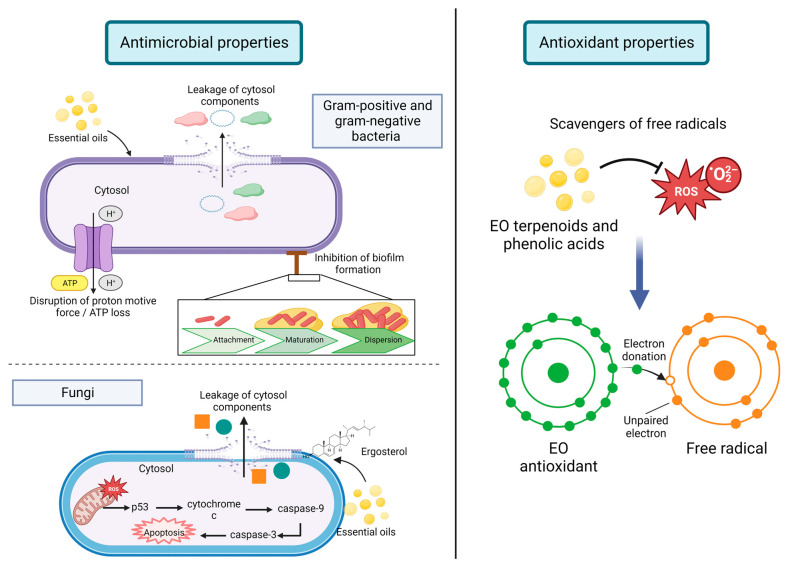
Mechanisms of action of antimicrobial and antioxidant properties of EO.

There is active research on in vitro study of the antimicrobial and antioxidant activities of EO (Figure 2). In the following sections, some studies are presented as novel developments in incorporating EO in different food systems [80].

Active packaging aims to extend food shelf-life and maintain or improve the packaged food’s properties. Unlike conventional food packaging, active packaging interacts with the product by incorporating compounds that could be released into the food or absorb substances responsible for the deterioration of the product. Incorporating EOs and their components into food packaging has been reported to increase food shelf-life since they have exhibited antimicrobial and antioxidant activities that can eradicate the presence of pathogen microorganisms and reduce lipid oxidation. Hence, EOs are an alternative to reduce or replace synthetic additives [10].

### 5.1. Antimicrobial Properties

Several studies demonstrated the antimicrobial activity of EO in food matrices. EO compounds can disrupt the bacterial membrane, damage their metabolic pathways, and prevent the synthesis of bacterial toxins [71]. Nevertheless, Gram-positive bacteria are more vulnerable to attaining antimicrobial effects, since hydrophobic molecules can easily pass through the cell membrane. In contrast, the outer membrane of Gram-negative bacteria can act as a barrier due to lipopolysaccharides’ presence, so they are more resistant toward hydrophobic compounds like those presented in EO; however, some phenolic compounds present in EO (i.e., thymol, eugenol, and carvacrol) can interfere with the cell wall outer membrane [81,82].

Due to their low molecular weight and lipophilic properties, EO can easily pass through the cell membrane and can inhibit bacteria growth by disrupting cell membranes, enzyme systems, and cell division, preventing biofilm formation, inducing bacterial membrane to produce clumps and auto-aggregation, hyperpolarization of cell membrane, altering lipid profile by formation of fatty acid hydroperoxidases caused by the oxygenation of unsaturated fatty acids within the cytosol, and by formation of cell membrane channels which cause leakage of ions, cellular material, and nucleic acids. Cell damage can lead to disruption of proton motive force and can cause ATP loss or affect ATP synthesis, changing the conformation of ATPase and inhibiting the expression of ATPase-related subunits interfere [82,83].

For instance, it has been reported that *Mentha* species contain hydrogen peroxide which can damage biomolecules of microorganisms, such as proteins, lipids, nucleic acids, and carbohydrates [84].

In terms of antimicrobial properties related to fungi, it has been reported that interaction of cinnamaldehyde, a major compound of cinnamon oil, with *Aspergillus flavus* caused elevated Ca^2+^ and ROS, decrease in mitochondrial membrane potential, release of cytochrome c, activation of metacaspase, and DNA damage. This compound increased the expression levels of apoptosis-related genes [82]. Moreover, EO exert antifungal effects through cell wall disruption causing leakage of cellular contents; it is thought that this disruption is caused by interactions of EO with ergosterol, which is the principal sterol present in fungi cell membranes and which controls permeability and fluidity [85].

Nonetheless, instead of synthetic antibiotics, EOs are commonly used in food systems as natural antiseptics to ensure food safety. The in vitro antimicrobial activity of particular EOs, such as oregano and thyme, against many Gram-positive and Gram-negative bacteria, yeasts, and molds has been thoroughly analyzed and documented [86]. For example, Siroli et al. [87] evaluated the efficacy of oregano and thyme essential oils for lamb’s lettuce decontamination and compared it to the efficacy of chlorine. The results showed that by applying EO, a product shelf-life similar to that obtained with chlorine was achieved [86].

Some studies reported by Ribeiro-Santos et al. [9] demonstrated that low-density polyethylene films with linalool and methyl chavicol exhibited antimicrobial activities against *Escherichia coli* and *Listeria innocua* in Cheddar cheese packaging previously inoculated with those organisms. Moreover, another polyethylene film with cinnamon EO and cinnamaldehyde, inhibited the growth of fungi (*Penicillium islandicum*, *Penicillium roqueforti*, *Penicillium nalgiovense*, *Eurotium repens*, *Aspergillus flavus*, *Candida albicans*, *Debaryomyces hansenii*, and *Zigosaccharomyces rouxii*) and bacteria (*Bacillus cereus*) at 4% (*w*/*w*) of active compounds. In comparison, *Listeria monocytogenes* and *Staphylococcus aureus* were inhibited at 8% (*w*/*w*), and *E. coli*, *Yersinia enterocolitica*, *Salmonella choleraesuise*, and *Pseudomonas aeruginosa* at >10% (*w*/*w*).

Furthermore, Masyita et al. [75] mentioned that *L. monocytogenes* is one of the major pathogens responsible for diseases in humans and animals. They reported a study in which clove and cinnamon EOs were evaluated in ground beef. Results demonstrated that 10% clove EO could decrease the growth of *L. monocytogenes*. Additionally, it has been reported that eucalyptus EO reduces *Saccharomyces cerevisiae* in Orangina juice.

### 5.2. Antioxidant Capacity

Antioxidant capacity exerted by EO is due to the double bonds present in alcohols, ethers, ketones, aldehydes, and phenolic compounds. Both terpenoid and phenylpropanoid families compromise phenolic compounds; these have high reactivity with peroxyl radicals, which are disposed of by hydrogen-atom transfer [83,88]. In the case of food products, lipid oxidation is the primary source of oxidation which produces rancidity; the use of EO polyphenolic compounds (i.e., terpenoids and phenolic acids) can act as oxygen and free radical scavengers to reduce lipid oxidation [83].

Some EO have been reported to exert in vitro antioxidant capacity. Routinary assays are used to measure radical scavenging properties against 1,1-diphenyl-2-picrylhydrazyl radical (DPPH) and 2,2′-azinobis(3-ethylbenzothiazoline)-6-sulfonic acid radical cation (ABTS). These methods are commonly used in the food industry to evaluate the antioxidant activity of specific compounds within the food matrix.

Radünz et al. [49] evaluated the effect of clove, thyme, oregano, and sweet orange EOs using DPPH assay. Results exhibited that clove EO had the highest inhibition percentage (94.3%). The primary compound found in clove EO was eugenol, to which authors attributed the remarkable ability to interact with free radicals compared to the major components of the other EO evaluated.

For instance, wild mint (*Mentha longifolia* var. *calliantha*) was evaluated by Asghari et al. [58] using DPPH and ABTS radicals to determine the free radical scavenging ability of the EO. It was demonstrated that wild mint EO had moderate antiradical potential in DPPH assay (5.8 mmol TEs/g oil), in contrast to a very high potential in ABTS assay (186 mmol TEs/g oil), expressed as equivalents of standard antioxidant compound Trolox (TEs). It is thought that this strong antioxidant capacity is due to the principal monoterpenes present in the EO, such as 1,8-cineol, linalool, and carvacrol, which are capable of donating hydrogens.

Moreover, two different species of oregano were evaluated by Sarikurkcu et al. [62], *Origanum vulgare* subsp. *vulgare* and subsp. *hirtum*. Besides performing DPPH and ABTS methods to measure radical scavenging, the β-carotene bleaching method was used to measure lipid peroxidation inhibition. In this assay, β-carotene is oxidized by radicals formed by linoleic acid oxidation in an emulsion, in which, eventually, the system loses its chromophore and is monitored spectrophotometrically. The results of this study exhibited that *O. vulgare* subsp. *vulgare* obtained 57.23 mg TEs/g oil for DPPH and 176.41 mg TEs/g oil for ABTS. This species exerted higher activity than that of *O. vulgare* subsp. *hirtum* in both assays. Due to differences in the chemical profile, the free radical capacity is influenced by the major components of the EO; in this regard, *O. vulgare* subsp. *vulgare* has a higher concentration of thymol and carvacrol (58.31 and 16.11%, respectively), which have been reported to be efficient scavengers of free radicals, as reported by Asghari et al. [58]. In terms of the β-carotene bleaching method, it obtained similar results since *O. vulgare* subsp. *vulgare* is the better inhibitor of linoleic acid oxidation (99.89%) compared to *O. vulgare* subsp. *hirtum* (23.54%). Hence, *O. vulgare* subsp. *vulgare* can be helpful for the management of lipid oxidation in the food industry.

Regarding the application of EOs as antioxidants in food products, there is active research in the meat industry to prevent oxidation reactions in meat and meat products. Studies reported by Pateiro et al. [89] suggested that oregano EO added at a concentration of 3% *w*/*w* significantly reduced oxidation reactions in raw and cooked porcine and bovine ground meat., Sage EO was also evaluated in fresh pork sausages, and there was a protective effect against lipid oxidation; furthermore, it had a higher antioxidant value than synthetic BHT. However, these antioxidant properties can be converted into prooxidants at high concentrations. An example of this was a study in which 150 ppm of rosemary was added to meat, and inhibition of lipid and protein oxidation was achieved. However, at 300 and 600 ppm concentrations, it promoted oxidation reactions because of interactions with fatty acids or concentration of tocopherols present in the product. Hence, it is essential to consider doses and meat matrix components interactions since their activity depends on the concentration utilized.

## 6. Stability and Formulation of EO

In active packaging, EOs are recommended to be introduced as micro or nanoemulsions. Incorporating EOs as micro or nanoemulsions prevents intense aroma [75]. In this regard, several strategies exist to incorporate EO into packaging materials. These techniques include a direct addition into polymeric materials, incorporation into coatings, immobilization with substrates, trapping into physical carries, insertion into headspace, or micro/nanoencapsulation in carriers, followed by incorporation into food matrices [90].

EOs are quite volatile and sensitive in certain conditions of illumination, temperature, and humidity, which are common during food processing. Therefore, researchers have used various encapsulation systems with different shell materials to protect EO from volatilization, oxidation, instability, and insolubility. The most common and effective encapsulation systems include liposomes, chitosan nanoparticles, cyclodextrin, silicon dioxide, nanoemulsions, solid lipid nanoparticles, nanofibers, and edible films. Furthermore, certain packaging methods, including food wraps and nanofibers, have also been proven to protect EO [91].

Recently, Reis et al. [92] published a review that addressed the conventional and most innovative encapsulation methods and the most relevant shell materials used in food systems. For instance, some essential factors have to be considered beforehand when selecting an appropriate encapsulation technique, such as desired particle size, shell materials’ physical properties, the core material’s solubility, controlled release, layer permeability, and costs. In this way, wall materials have to accomplish three different stages in order to be considered successful: (i) formation of a wall around the core; (ii) core components have to be kept inside the capsule without any release or degradation; and (iii) incorporation of the capsule in food systems and correct release of oil components. Encapsulation techniques are divided into two different categories: physical and chemical methods. Physical methods do not involve polymerization reactions, and microcapsule formation occurs mechanically. This classification includes extrusion, fluidization, lyophilization, solvent removal, spray drying, and supercritical fluid techniques. For its part, chemical methods involve polymerization reactions, and techniques include coacervation, ionic gelation, liposomes, and miniemulsion polymerization.

**Figure 3:**
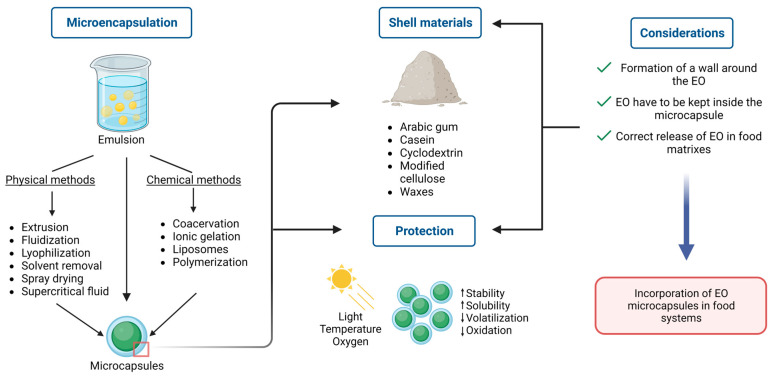
Schematic representation of encapsulation process and common shell materials used in food systems.

Moreover, common shell materials used in food matrixes include polysaccharides (i.e., starch, dextrin, maltodextrin, modified starch, cyclodextrin, chitosan), gums (i.e., arabic gum, sodium alginate), proteins (i.e., whey, soy, casein, gelatin), cellulose (i.e., modified cellulose), and lipids (i.e., waxes, paraffin, fats) (Figure 3). There is a discussion about emulsification as an encapsulation technique or as a step before encapsulation since the latter guarantees an improvement in storage stability because droplets are immobilized in a solid matrix. At the same time, the former comprises a liquid wall material that could disfavor the core compounds’ physical and chemical resistance and retention. Nevertheless, emulsification methods are divided into conventional methods (i.e., colloid mill, high-speed mixer, high-pressure homogenizer, ultrasonic homogenizer) and membrane emulsification methods. Some advantages regarding the membrane emulsification method as a novel technique are lower energy demand, low shear rates, lower temperature elevation, more control of droplet size, and ease of scaling up. However, some disadvantages of this method are fouling phenomena on the membrane surface and pores, the membrane’s short lifetime, and more resistance in mass transfer regarding the membrane.

Research has shown that nanoencapsulation boosts the preservative potential of plant essential oils in vitro and in food systems. For instance, Jamil et al. [93] investigated the antimicrobial efficacy of cardamom oil encapsulated in chitosan-based nanoparticles. The results demonstrated that the nano-encapsulated EO exhibits excellent antimicrobial potential against *Escherichia coli* and *Staphylococcus aureus* [94]. This encapsulation system has been used to protect EO effectively and improve their functional performance in food systems [95]. Amiri et al. [96] used conventional nanoemulsion and fortified nanoemulsion as delivery systems for *Zataria multiflora* in corn starch and analyzed its effect on the sensory properties of ground beef patties. The results showed that the fortified nanoemulsion had the highest scores for all the sensory parameters, while the control showed the lowest scores [95]. Another study by Viacava et al. [97] also analyzed the effect nanoencapsulation has on sensory attributes. The researchers studied the impact of free and β-cyclodextrin encapsulated thyme EO on the quality of minimally processed Romaine lettuce. The results demonstrated that the lettuce treated with the nano-encapsulated EO exhibited better organoleptic quality scores than the control and free EO-treated lettuce [95]. Therefore, the authors concluded that nanoencapsulation could help improve the organoleptic attributes of food items.

Other delivery systems, including surfactant-based systems, films, fibers, and oleogels, are being investigated for their effectiveness. For instance, Chen & Yang [98] have used Quillaja saponin, a natural triterpene, to stabilize orange oil via oleogel [99]. The authors concluded that the oleogels with high gel strength had good thixotropic recovery and reversibility to reconstituted emulsions. Therefore, oleogels show great potential for food, cosmetics, and pharmaceutical applications [99]. Quillaja saponin has also been used by Sedaghat Doost et al. [100] to stabilize thymol nanoemulsions. With this system, the authors could create thymol emulsions with long-term stability that contained a relatively low content of green solvents.

Furthermore, the authors reported that, compared to free thymol, emulsification improved its antioxidant activity. Another innovative strategy has been investigated by Silva et al. [101], as they encapsulated coriander essential oil in cyclodextrin nanosponges to achieve a controlled oil release. The authors reported that cyclodextrin polymers can effectively incorporate and release coriander essential oil and that including this oil inside the nanosponge improves the crystallinity of the polymer, which leads to a more effective controlled release.

For instance, in a study performed by Siahbalaei et al. [54], some EO (*Oliveria decumbens*, *Thymus kotschyanus*, *Trachyspermum ammi*, and *Zataria multiflora*) were individually encapsulated into a gelatin-pectin nanocomposite consisting of gelatin (7 g), pectin (3 g), 100 mL acetic acid (60%), glycerol (100 mg/g of total polymer), and glutaraldehyde (10 mg/g of total polymer), resulting in a 500 to 700 nm size range. Each composite displayed several bioactivities such as glucose autoxidation inhibition, lipid peroxidation inhibition, protein oxidation, glycation inhibition, and α-amylase and α-glucosidase inhibition activity.

## 7. Conclusions

EOs have been demonstrated to have anti-obesity, antidiabetic activities, and neuroprotective effects. They can be used as a natural alternative to treat these disorders even though further investigation is needed to elucidate the interaction between EO and metabolic pathways involved in developing these diseases at both the cellular level and in living organisms.

EO are an attractive alternative for substituting synthetic additives since they show antimicrobial and antioxidant activities that extend food products’ shelf-life and guarantee food safety to consumers. EO micro/nanoencapsulation is a novel technological approach for their stabilization and incorporation into different food systems. Nevertheless, the interaction of these compounds with food matrices needs to be studied in detail before their incorporation to obtain the expected results.

The scientific information herein presented demonstrates the dual role of EO in preventing and treating metabolic syndrome-related disorders and their well-demonstrated role as antioxidant and antimicrobial food additives. This dual role makes EO excellent candidates to formulate dietary supplements and functional foods.

Over the course of this review, there are several research gaps and perspectives in the field that have been identified and need to be further explored. The first of these is that there are several factors, with respect to the raw material used, that must be required when carrying out in vitro or in vivo studies. Among them are the quality, the extraction method, the part of the plant used, and its stage of maturation. Since, depending on this, primary compounds may vary, so too will bioactive properties be impacted as well.

Another point to consider is the nanoencapsulation of EO. Besides preserving and/or enhancing bioactivity, it needs to ensure a benefit in the organoleptic properties of food systems, such as flavor, color, aroma, and texture, so that in this way, a high consumption of functional foods fortified with EO is guaranteed.

Furthermore, studies about synergistic combinations of EO to exert therapeutical and technological properties in food systems need to be performed, especially regarding the quality and quantity used of each one in food matrixes, in order to offer the promised bioactivity. In this regard, novel technological approaches to enhance EO stability in food systems deserve more research to scale up processes in an industrial manner and, in this way, to overcome current health problems.

## Data Availability

Not applicable.

## References

[1] Yen H.-F., Hsieh C.-T., Hsieh T.-J., Chang F.-R., Wang C.-K. (2015). In vitro anti-diabetic effect and chemical component analysis of 29 essential oils products. J. Food Drug Anal..

[2] Santana-Gálvez J., Cisneros-Zevallos L., Jacobo-Velázquez D.A. (2017). Chlorogenic Acid: Recent Advances on Its Dual Role as a Food Additive and a Nutraceutical against Metabolic Syndrome. Molecules.

[3] Shenoy A., Buttar H.S., Dicholkar P., Kaur G., Chintamaneni M., Singh R.B., Watanabe S., Isaza A.A. (2022). Chapter 39-Role of nutraceuticals, functional foods, and spices in the management of metabolic syndrome and related disorders. Functional Foods and Nutraceuticals in Metabolic and Non-Communicable Diseases.

[4] Nijhawan P., Behl T. (2020). Nutraceuticals in the management of obesity. Obes. Med..

[5] Bakkali F., Averbeck S., Averbeck D., Idaomar M. (2008). Biological effects of essential oils—A review. Food Chem. Toxicol. Int. J. Publ. Br. Ind. Biol. Res. Assoc..

[6] Unusan N. (2020). Essential oils and microbiota: Implications for diet and weight control. Trends Food Sci. Technol..

[7] Russo R., Corasaniti M.T., Bagetta G., Morrone L.A. (2015). Exploitation of Cytotoxicity of Some Essential Oils for Translation in Cancer Therapy. Evid.-Based Complement. Altern. Med..

[8] Majid I., Khan S., Aladel A., Dar A.H., Adnan M., Khan M.I., Awadelkareem A.M., Ashraf S.A. (2023). Recent insights into green extraction techniques as efficient methods for the extraction of bioactive components and essential oils from foods. CyTA-J. Food.

[9] Pelvan E., Karaoğlu Ö., Fırat E.Ö., Kalyon K.B., Ros E., Alasalvar C. (2022). Immunomodulatory effects of selected medicinal herbs and their essential oils: A comprehensive review. J. Funct. Foods.

[10] Ribeiro-Santos R., Andrade M., de Melo N.R., Sanches-Silva A. (2017). Use of essential oils in active food packaging: Recent advances and future trends. Trends Food Sci. Technol..

[11] Rochlani Y., Pothineni N.V., Kovelamudi S., Mehta J.L. (2017). Metabolic syndrome: Pathophysiology, management, and modulation by natural compounds. Ther. Adv. Cardiovasc. Dis..

[12] Zhang K., Sun J., Fan M., Qian H., Ying H., Li Y., Wang L. (2021). Functional ingredients present in whole-grain foods as therapeutic tools to counteract obesity: Effects on brown and white adipose tissues. Trends Food Sci. Technol..

[13] Bayliak M.M., Dmytriv T.R., Melnychuk A.V., Strilets N.V., Storey K.B., Lushchak V.I. (2021). Chamomile as a potential remedy for obesity and metabolic syndrome. EXCLI J..

[14] Spalletta S., Flati V., Toniato E., Di Gregorio J., Marino A., Pierdomenico L., Marchisio M., D’Orazi G., Cacciatore I., Robuffo I. (2018). Carvacrol reduces adipogenic differentiation by modulating autophagy and ChREBP expression. PLoS ONE.

[15] Hong S.J., Cho J., Boo C.G., Youn M.Y., Pan J.H., Kim J.K., Shin E.-C. (2020). Inhalation of Patchouli (*Pogostemon Cablin* Benth.) Essential Oil Improved Metabolic Parameters in Obesity-Induced Sprague Dawley Rats. Nutrients.

[16] Rutkowski J.M., Stern J.H., Scherer P.E. (2015). The cell biology of fat expansion. J. Cell Biol..

[17] Ghaben A.L., Scherer P.E. (2019). Adipogenesis and metabolic health. Nat. Rev. Mol. Cell Biol..

[18] Ngamdokmai N., Paracha T.U., Waranuch N., Chootip K., Wisuitiprot W., Suphrom N., Insumrong K., Ingkaninan K. (2021). Effects of Essential Oils and Some Constituents from Ingredients of Anti-Cellulite Herbal Compress on 3T3-L1 Adipocytes and Rat Aortae. Pharmaceuticals.

[19] Lee M.-H., Chen Y.-Y., Tsai J.-W., Wang S.-C., Watanabe T., Tsai Y.-C. (2011). Inhibitory effect of β-asarone, a component of Acorus calamus essential oil, on inhibition of adipogenesis in 3T3-L1 cells. Food Chem..

[20] Hwang D.I., Won K.-J., Kim D.-Y., Yoon S.W., Park J.-H., Kim B., Lee H.M. (2016). Anti-adipocyte Differentiation Activity and Chemical Composition of Essential Oil from *Artemisia annua*. Nat. Prod. Commun..

[21] Ko H.-S., Lee H.-J., Sohn E.J., Yun M., Lee M.-H., Kim S.-H. (2013). Essential Oil of *Pinus koraiensis* Exerts Antiobesic and Hypolipidemic Activity via Inhibition of Peroxisome Proliferator-Activated Receptors Gamma Signaling. Evid.-Based Complement. Altern. Med..

[22] Cheng B.-H., Sheen L.-Y., Chang S.-T. (2018). Hypolipidemic effects of S -(+)-linalool and essential oil from Cinnamomum osmophloeum ct. linalool leaves in mice. J. Tradit. Complement. Med..

[23] Sprenger S., Woldemariam T., Kotchoni S., Elshabrawy H.A., Chaturvedi L.S. (2022). Lemongrass essential oil and its major constituent citral isomers modulate adipogenic gene expression in 3T3-L1 cells. J. Food Biochem..

[24] Lai Y.-S., Chen W.-C., Ho C.-T., Lu K.-H., Lin S.-H., Tseng H.-C., Lin S.-Y., Sheen L.-Y. (2014). Garlic Essential Oil Protects against Obesity-Triggered Nonalcoholic Fatty Liver Disease through Modulation of Lipid Metabolism and Oxidative Stress. J. Agric. Food Chem..

[25] Lai Y.-S., Lee W.-C., Lin Y.-E., Ho C.-T., Lu K.-H., Lin S.-H., Panyod S., Chu Y.-L., Sheen L.-Y. (2016). Ginger Essential Oil Ameliorates Hepatic Injury and Lipid Accumulation in High Fat Diet-Induced Nonalcoholic Fatty Liver Disease. J. Agric. Food Chem..

[26] Asnaashari S., Delazar A., Habibi B., Vasfi R., Nahar L., Hamedeyazdan S., Sarker S.D. (2010). Essential Oil from *Citrus aurantifolia* prevents ketotifen-induced weight-gain in mice. Phytother. Res..

[27] Ciftci M., Simsek U.G., Dalkilic B., Erisir Z., Mutlu S.I., Azman M.A., Ozcelik M., Yilmaz O., Tonbak F. (2018). Effects of Essential Oil Mixture Supplementation to Basal Diet on Fattening Performance, Blood Parameters and Antioxidant Status of Tissues in Japanese Quails Exposed to Low Ambient Temperature. J. Anim. Plant Sci..

[28] Taghizadeh M., Memarzadeh M.R., Asemi Z., Esmaillzadeh A. (2015). Effect of the cumin cyminum L. Intake on Weight Loss, Metabolic Profiles and Biomarkers of Oxidative Stress in Overweight Subjects: A Randomized Double-Blind Placebo-Controlled Clinical Trial. Ann. Nutr. Metab..

[29] Li D., Wu H., Dou H. (2019). Weight loss effect of sweet orange essential oil microcapsules on obese SD rats induced by high-fat diet. Biosci. Biotechnol. Biochem..

[30] Batubara I., Suparto I.H., Sa’Diah S., Matsuoka R., Mitsunaga T. (2015). Effects of Inhaled Citronella Oil and Related Compounds on Rat Body Weight and Brown Adipose Tissue Sympathetic Nerve. Nutrients.

[31] Shen J., Niijima A., Tanida M., Horii Y., Maeda K., Nagai K. (2005). Olfactory stimulation with scent of grapefruit oil affects autonomic nerves, lipolysis and appetite in rats. Neurosci. Lett..

[32] Goyal R., Jialal I. (2022). Diabetes Mellitus Type 2. StatPearls.

[33] Kharroubi A.T., Darwish H.M. (2015). Diabetes mellitus: The epidemic of the century. World J. Diabetes.

[34] Blaslov K., Naranđa F.S., Kruljac I., Renar I.P. (2018). Treatment approach to type 2 diabetes: Past, present and future. World J. Diabetes.

[35] Galicia-Garcia U., Benito-Vicente A., Jebari S., Larrea-Sebal A., Siddiqi H., Uribe K., Ostolaza H., Martín C. (2020). Pathophysiology of Type 2 Diabetes Mellitus. Int. J. Mol. Sci..

[36] Blaak E.E., Antoine J.-M., Benton D., Björck I., Bozzetto L., Brouns F., Diamant M., Dye L., Hulshof T., Holst J.J. (2012). Impact of postprandial glycaemia on health and prevention of disease. Obes. Rev..

[37] Aisa H.A., Gao Y., Yili A., Ma Q., Cheng Z., Watson R.R., Preedy V.R. (2019). Beneficial Role of Chickpea (Cicer arietinum L.) Functional Factors in the Intervention of Metabolic Syndrome and Diabetes Mellitus. Bioactive Food as Dietary Interventions for Diabetes.

[38] Hiyoshi T., Fujiwara M., Yao Z. (2019). Postprandial hyperglycemia and postprandial hypertriglyceridemia in type 2 diabetes. J. Biomed. Res..

[39] Beręsewicz A., Preedy V.R. (2020). NADPH oxidases, nuclear factor kappa B, NF-E2-related factor2, and oxidative stress in diabetes. Diabetes, Oxidative Stress and Dietary Antioxidants.

[40] Yang Y., Zhang J.-L., Shen L.-H., Feng L.-J., Zhou Q. (2021). Inhibition mechanism of diacylated anthocyanins from purple sweet potato (*Ipomoea batatas* L.) against α-amylase and α-glucosidase. Food Chem..

[41] Zheng Y., Yang W., Sun W., Chen S., Liu D., Kong X., Tian J., Ye X. (2020). Inhibition of porcine pancreatic α-amylase activity by chlorogenic acid. J. Funct. Foods.

[42] Warren F.J., Zhang B., Waltzer G., Gidley M.J., Dhital S. (2015). The interplay of α-amylase and amyloglucosidase activities on the digestion of starch in in vitro enzymic systems. Carbohydr. Polym..

[43] Artasensi A., Pedretti A., Vistoli G., Fumagalli L. (2020). Type 2 Diabetes Mellitus: A Review of Multi-Target Drugs. Molecules.

[44] Habtemariam S. (2017). Antidiabetic Potential of Monoterpenes: A Case of Small Molecules Punching above Their Weight. Int. J. Mol. Sci..

[45] Hanefeld M., Mertes G., Huhtaniemi I., Martini L. (2019). Treatment: Alpha Glucosidase Inhibitors. Encyclopedia of Endocrine Diseases.

[46] Liu Z., Zhao X., Sun W., Wang Y., Liu S., Kang L. (2017). Metformin combined with acarbose vs. single medicine in the treatment of type 2 diabetes: A meta-analysis. Exp. Ther. Med..

[47] Al-Asri J., Fazekas E., Lehoczki G., Perdih A., Görick C., Melzig M.F., Gyémánt G., Wolber G., Mortier J. (2015). From carbohydrates to drug-like fragments: Rational development of novel α-amylase inhibitors. Bioorg. Med. Chem..

[48] Leyva-López N., Gutiérrez-Grijalva E.P., Vazquez-Olivo G., Heredia J.B. (2017). Essential Oils of Oregano: Biological Activity beyond Their Antimicrobial Properties. Mol. J. Synth. Chem. Nat. Prod. Chem..

[49] Radünz M., Camargo T.M., dos Santos Hackbart H.C., Alves P.I.C., Radünz A.L., Gandra E.A., Zavareze E.D.R. (2021). Chemical composition and in vitro antioxidant and antihyperglycemic activities of clove, thyme, oregano, and sweet orange essential oils. LWT.

[50] Rahali N., Mehdi S., Younsi F., Boussaid M., Messaoud C. (2017). Antioxidant, α-amylase, and acetylcholinesterase inhibitory activities of *Hertia cheirifolia* essential oils: Influence of plant organs and seasonal variation. Int. J. Food Prop..

[51] Pavlić B., Teslić N., Zengin G., Đurović S., Rakić D., Cvetanović A., Gunes A., Zeković Z. (2021). Antioxidant and enzyme-inhibitory activity of peppermint extracts and essential oils obtained by conventional and emerging extraction techniques. Food Chem..

[52] Chung M.J., Cho S.-Y., Bhuiyan M.J.H., Kim K.H., Lee S.-J. (2010). Anti-diabetic effects of lemon balm (*Melissa officinalis*) essential oil on glucose- and lipid-regulating enzymes in type 2 diabetic mice. Br. J. Nutr..

[53] Jaradat N., Al-Maharik N., Abdallah S., Shawahna R., Mousa A., Qtishat A. (2020). Nepeta curviflora essential oil: Phytochemical composition, antioxidant, anti-proliferative and anti-migratory efficacy against cervical cancer cells, and α-glucosidase, α-amylase and porcine pancreatic lipase inhibitory activities. Ind. Crop. Prod..

[54] Siahbalaei R., Kavoosi G., Shakeri R. (2020). In vitro antioxidant and antidiabetic activity of essential oils encapsulated in gelatin-pectin particles against sugar, lipid and protein oxidation and amylase and glucosidase activity. Food Sci. Nutr..

[55] Oboh G., Olasehinde T.A., Ademosun A.O. (2017). Inhibition of enzymes linked to type-2 diabetes and hypertension by essential oils from peels of orange and lemon. Int. J. Food Prop..

[56] Oboh G., Akinbola I.A., Ademosun A.O., Sanni D.M., Odubanjo O.V., Olasehinde T.A., Oyeleye S.I. (2015). Essential Oil from Clove Bud (*Eugenia aromatica* Kuntze) Inhibit Key Enzymes Relevant to the Management of Type-2 Diabetes and Some Pro-oxidant Induced Lipid Peroxidation in Rats Pancreas in vitro. J. Oleo Sci..

[57] Belhadj S., Hentati O., Hammami M., Ben Hadj A., Boudawara T., Dammak M., Zouari S., El Feki A. (2018). Metabolic impairments and tissue disorders in alloxan-induced diabetic rats are alleviated by *Salvia officinalis* L. essential oil. Biomed. Pharmacother..

[58] Asghari B., Zengin G., Bahadori M.B., Abbas-Mohammadi M., Dinparast L. (2018). Amylase, glucosidase, tyrosinase, and cholinesterases inhibitory, antioxidant effects, and GC-MS analysis of wild mint (*Mentha longifolia* var. *calliantha*) essential oil: A natural remedy. Eur. J. Integr. Med..

[59] Loizzo M., Saab A., Statti G., Menichini F. (2007). Composition and α-amylase inhibitory effect of essential oils from *Cedrus libani*. Fitoterapia.

[60] Oboh G., Ademosun A.O., Odubanjo O.V., Akinbola I.A. (2013). Antioxidative Properties and Inhibition of Key Enzymes Relevant to Type-2 Diabetes and Hypertension by Essential Oils from Black Pepper. Adv. Pharmacol. Sci..

[61] Sebai H., Selmi S., Rtibi K., Souli A., Gharbi N., Sakly M. (2013). Lavender (*Lavandula stoechas* L.) essential oils attenuate hyperglycemia and protect against oxidative stress in alloxan-induced diabetic rats. Lipids Health Dis..

[62] Sarikurkcu C., Zengin G., Oskay M., Uysal S., Ceylan R., Aktumsek A. (2015). Composition, antioxidant, antimicrobial and enzyme inhibition activities of two Origanum vulgare subspecies (subsp. *vulgare* and subsp. *hirtum*) essential oils. Ind. Crop. Prod..

[63] Aumeeruddy-Elalfi Z., Lall N., Fibrich B., van Staden A.B., Hosenally M., Mahomoodally M.F. (2018). Selected essential oils inhibit key physiological enzymes and possess intracellular and extracellular antimelanogenic properties in vitro. J. Food Drug Anal..

[64] Ding W., Liping N., Xing H., Wei Z., Zhoua Q., Nong R., Chen J. (2020). Essential oil extracted from leaf of *Phoebe bournei* (Hemsl.) yang: Chemical constituents, antitumor, antibacterial, hypoglycemic activities. Nat. Prod. Res..

[65] Selmi S., Rtibi K., Grami D., Sebai H., Marzouki L. (2017). Rosemary (*Rosmarinus officinalis*) essential oil components exhibit anti-hyperglycemic, anti-hyperlipidemic and antioxidant effects in experimental diabetes. Pathophysiology.

[66] Mosbah H., Chahdoura H., Kammoun J., Hlila M.B., Louati H., Hammami S., Flamini G., Achour L., Selmi B. (2018). *Rhaponticum acaule* (L) DC essential oil: Chemical composition, in vitro antioxidant and enzyme inhibition properties. BMC Complement. Altern. Med..

[67] Agatonovic-Kustrin S., Kustrin E., Morton D.W. (2019). Essential oils and functional herbs for healthy aging. Neural Regen. Res..

[68] Ezeorba T.P.C., Chukwudozie K.I., Ezema C.A., Anaduaka E.G., Nweze E.J., Okeke E.S. (2022). Potentials for health and therapeutic benefits of garlic essential oils: Recent findings and future prospects. Pharmacol. Res.-Mod. Chin. Med..

[69] García-Ayllón M.-S., Small D.H., Avila J., Saez-Valero J. (2011). Revisiting the role of acetylcholinesterase in Alzheimer’s disease: Cross-talk with P-tau and β-amyloid. Front. Mol. Neurosci..

[70] Bouayed J., Bohn T. (2012). Nutrition, Well-Being and Health.

[71] Horky P., Skalickova S., Smerkova K., Skladanka J. (2019). Essential Oils as a Feed Additives: Pharmacokinetics and Potential Toxicity in Monogastric Animals. Animals.

[72] Kohlert C., van Rensen I., März R., Schindler G., Graefe E.U., Veit M. (2000). Bioavailability and Pharmacokinetics of Natural Volatile Terpenes in Animals and Humans. Planta Med..

[73] Sherwood L. (2012). Human Physiology: From Cells to Systems.

[74] Waxenbaum J.A., Reddy V., Varacallo M. (2022). Anatomy, Autonomic Nervous System. StatPearls.

[75] Masyita A., Sari R.M., Astuti A.D., Yasir B., Rumata N.R., Emran T.B., Nainu F., Simal-Gandara J. (2022). Terpenes and terpenoids as main bioactive compounds of essential oils, their roles in human health and potential application as natural food preservatives. Food Chem. X.

[76] Cal K. (2006). Skin Penetration of Terpenes from Essential Oils and Topical Vehicles. Planta Med..

[77] Herman A., Herman A.P. (2015). Essential oils and their constituents as skin penetration enhancer for transdermal drug delivery: A review. J. Pharm. Pharmacol..

[78] Jiang Q., Wu Y., Zhang H., Liu P., Yao J., Yao P., Chen J., Duan J. (2017). Development of essential oils as skin permeation enhancers: Penetration enhancement effect and mechanism of action. Pharm. Biol..

[79] Varman R.M., Singh S. (2012). Investigation of Effects of Terpene Skin Penetration Enhancers on Stability and Biological Activity of Lysozyme. AAPS PharmSciTech.

[80] Sendra E. (2016). Essential Oils in Foods: From Ancient Times to the 21st Century. Foods.

[81] Hyldgaard M., Mygind T., Meyer R.L. (2012). Essential Oils in Food Preservation: Mode of Action, Synergies, and Interactions with Food Matrix Components. Front. Microbiol..

[82] Li Y.-X., Erhunmwunsee F., Liu M., Yang K., Zheng W., Tian J. (2022). Antimicrobial mechanisms of spice essential oils and application in food industry. Food Chem..

[83] Jackson-Davis A., White S., Kassama L.S., Coleman S., Shaw A., Mendonca A., Cooper B., Thomas-Popo E., Gordon K., London L. (2023). A Review of Regulatory Standards and Advances in Essential Oils as Antimicrobials in Foods. J. Food Prot..

[84] Saqib S., Ullah F., Naeem M., Younas M., Ayaz A., Ali S., Zaman W. (2022). *Mentha*: Nutritional and Health Attributes to Treat Various Ailments Including Cardiovascular Diseases. Molecules.

[85] Mukurumbira A., Shellie R., Keast R., Palombo E., Jadhav S. (2022). Encapsulation of essential oils and their application in antimicrobial active packaging. Food Control.

[86] Patrignani F., Siroli L., Serrazanetti D.I., Gardini F., Lanciotti R. (2015). Innovative strategies based on the use of essential oils and their components to improve safety, shelf-life and quality of minimally processed fruits and vegetables. Trends Food Sci. Technol..

[87] Siroli L., Patrignani F., Serrazanetti D.I., Tabanelli G., Montanari C., Gardini F., Lanciotti R. (2015). Lactic acid bacteria and natural antimicrobials to improve the safety and shelf-life of minimally processed sliced apples and lamb’s lettuce. Food Microbiol..

[88] Amorati R., Foti M.C., Valgimigli L. (2013). Antioxidant Activity of Essential Oils. J. Agric. Food Chem..

[89] Pateiro M., Barba F.J., Domínguez R., Sant’Ana A.S., Khaneghah A.M., Gavahian M., Gómez B., Lorenzo J.M. (2018). Essential oils as natural additives to prevent oxidation reactions in meat and meat products: A review. Food Res. Int..

[90] Stoleru E., Brebu M. (2021). Stabilization Techniques of Essential Oils by Incorporation into Biodegradable Polymeric Materials for Food Packaging. Molecules.

[91] Zhu Y., Li C., Cui H., Lin L. (2021). Encapsulation strategies to enhance the antibacterial properties of essential oils in food system. Food Control.

[92] Reis D.R., Ambrosi A., Di Luccio M. (2022). Encapsulated essential oils: A perspective in food preservation. Future Foods.

[93] Jamil B., Abbasi R., Abbasi S., Imran M., Khan S.U., Ihsan A., Javed S., Bokhari H. (2016). Encapsulation of Cardamom Essential Oil in Chitosan Nano-composites: In-vitro Efficacy on Antibiotic-Resistant Bacterial Pathogens and Cytotoxicity Studies. Front. Microbiol..

[94] Prakash B., Kujur A., Yadav A., Kumar A., Singh P.P., Dubey N.K. (2018). Nanoencapsulation: An efficient technology to boost the antimicrobial potential of plant essential oils in food system. Food Control.

[95] Chaudhari A.K., Singh V.K., Das S., Dubey N.K. (2021). Nanoencapsulation of essential oils and their bioactive constituents: A novel strategy to control mycotoxin contamination in food system. Food Chem. Toxicol..

[96] Amiri E., Aminzare M., Azar H.H., Mehrasbi M.R. (2019). Combined antioxidant and sensory effects of corn starch films with nanoemulsion of *Zataria multiflora* essential oil fortified with cinnamaldehyde on fresh ground beef patties. Meat Sci..

[97] Viacava G.E., Ayala-Zavala J.F., González-Aguilar G.A., Ansorena M.R. (2018). Effect of free and microencapsulated thyme essential oil on quality attributes of minimally processed lettuce. Postharvest Biol. Technol..

[98] Chen X.-W., Yang X.-Q. (2019). Characterization of Orange Oil Powders and Oleogels Fabricated from Emulsion Templates Stabilized Solely by a Natural Triterpene Saponin. J. Agric. Food Chem..

[99] Doost A.S., Nasrabadi M.N., Kassozi V., Nakisozi H., Van der Meeren P. (2020). Recent advances in food colloidal delivery systems for essential oils and their main components. Trends Food Sci. Technol..

[100] Doost A.S., Van Camp J., Dewettinck K., Van der Meeren P. (2019). Production of thymol nanoemulsions stabilized using Quillaja Saponin as a biosurfactant: Antioxidant activity enhancement. Food Chem..

[101] Silva F., Caldera F., Trotta F., Nerín C., Domingues F.C. (2019). Encapsulation of coriander essential oil in cyclodextrin nanosponges: A new strategy to promote its use in controlled-release active packaging. Innov. Food Sci. Emerg. Technol..

